# Synaptic vesicle release during ribbon synapse formation of cone photoreceptors

**DOI:** 10.3389/fncel.2022.1022419

**Published:** 2022-11-04

**Authors:** Adam Davison, Kaspar Gierke, Johann Helmut Brandstätter, Norbert Babai

**Affiliations:** Division of Animal Physiology/Neurobiology, Department of Biology, Friedrich-Alexander-Universität Erlangen-Nürnberg, Erlangen, Germany

**Keywords:** development, retina, cone photoreceptor, ribbon synapse, synaptic vesicle release, pool size, calcium

## Abstract

Mammalian cone photoreceptors enable through their sophisticated synapse the high-fidelity transfer of visual information to second-order neurons in the retina. The synapse contains a proteinaceous organelle, called the synaptic ribbon, which tethers synaptic vesicles (SVs) at the active zone (AZ) close to voltage-gated Ca^2+^ channels. However, the exact contribution of the synaptic ribbon to neurotransmission is not fully understood, yet. In mice, precursors to synaptic ribbons appear within photoreceptor terminals shortly after birth as free-floating spherical structures, which progressively elongate and then attach to the AZ during the following days. Here, we took advantage of the process of synaptic ribbon maturation to study their contribution to SV release. We performed whole-cell patch-clamp recordings from cone photoreceptors at three postnatal (P) development stages (P8–9, P12–13, >P30) and measured evoked SV release, SV replenishment rate, recovery from synaptic depression, domain organization of voltage-sensitive Ca^2+^ channels, and Ca^2+^-sensitivity of exocytosis. Additionally, we performed electron microscopy to determine the density of SVs at ribbon-free and ribbon-occupied AZs. Our results suggest that ribbon attachment does not organize the voltage-sensitive Ca^2+^ channels into nanodomains or control SV release probability. However, ribbon attachment increases SV density at the AZ, increases the pool size of readily releasable SVs available for evoked SV release, facilitates SV replenishment without changing the SV pool refilling time, and increases the Ca^2+^- sensitivity of glutamate release.

## Introduction

Rod and cone photoreceptors are specialized sensory neurons that continuously transduce light-evoked, graded membrane potential (V_*m*_) changes across a broad range of light intensities (Choi et al., 2005). These light-evoked changes in V_*m*_ spread from the outer segment to the synaptic terminal and activate voltage-sensitive Ca^2+^ ion channels, which then trigger neurotransmitter release to second-order neurons, the bipolar and horizontal cells (Pangrsic et al., 2018; Davison et al., 2022a). In dark conditions, the membrane potential of photoreceptors is depolarized to ∼–40 mV. At this V_*m*_, Ca^2+^ flows through the voltage-sensitive Ca^2+^ channels, which are concentrated at the active zone (AZ), triggers tonic synaptic vesicle (SV) release (Dieck et al., 2005). Bright light causes membrane hyperpolarization to a level of ∼–70 mV, which deactivates the voltage-sensitive Ca^2+^ channels, thereby reducing SV release. This type of exocytosis is called spontaneous SV release (Cork et al., 2016; Grassmeyer et al., 2019). However, sudden V_*m*_ changes, which are caused by quick light to dark transitions, are able to generate transient, so-called evoked SV release events, in ribbon synapses (Singer and Diamond, 2003). These three types of SV release play a key role in coding light information at the “first step” in vision.

Photoreceptor synapses are structurally different from conventional synapses (Lankford et al., 2020). Their AZs possess a proteinaceous organelle called the synaptic ribbon. Synaptic ribbons extend from the AZs into the synaptic terminal and are surrounded by numerous tethered SVs. In photoreceptors, synaptic ribbons have a horseshoe−shaped appearance (Dieck and Brandstatter, 2006; Mercer and Thoreson, 2011). Photoreceptor terminals contain a large amount of SVs but only a small number of these SVs are prepared for immediate exocytosis. These fusion-competent SVs constitute the readily releasable pool (RRP) (Kaeser and Regehr, 2017). Electrophysiological measurements of the total number of SVs released during a step depolarization in cone photoreceptors reveal three distinct components (Bartoletti et al., 2010; Grabner et al., 2016; Thoreson et al., 2016). The first and fastest SV release component occurs within a few milliseconds of stimulation and matches with the number of SVs present at the bottom of the synaptic ribbon. The second, slower SV release component correlates to the number of SVs tethered further up on the synaptic ribbon. The third component of SV release maintains a steady rate, likely by the continuous replenishment of SVs. However, the exact contribution of the synaptic ribbon to the different modes of SV release is not fully understood.

Mouse cone photoreceptor ribbon synapse development primarily occurs in the first two postnatal weeks (Blanks et al., 1974; Davison et al., 2022b). During this time, synaptic protein constituents, such as ribeye, picollino, bassoon, ubMunc13-2, CAST, RIM, and the L-type Ca^2+^ channel α1 subunit arrive to the AZ (Hermes et al., 1992; Regus-Leidig et al., 2009). Starting from ∼P4, free-floating synaptic ribbons gradually anchor to the AZ. Mice open their eyes at ∼P12–13 but by this time, the synapses of the photoreceptors are not yet fully developed. For example, in cone photoreceptors, only 30% of all ribbons are anchored to the AZs at this age (Davison et al., 2022b). Consequently, synapse development continues until maturity, which is completed at ∼P30 (Vinberg et al., 2014; Bonezzi et al., 2018). Whilst numerous studies have described the structural development of the photoreceptors synapse, we have a limited understanding of its functional development.

We have recently conducted a study on the functional and structural maturation of mouse cone photoreceptor ribbon synapses (Davison et al., 2022b). We found that during postnatal development synaptic ribbons gradually occupy the ribbon-free AZs, mimicking the transition from a ribbon-free synapse to a ribbon-type synapse. Moreover, we found that the presence of synaptic ribbons at the AZ attenuates tonic SV release and aids multiquantal SV release. Building on these findings, the current study represents a logical continuation of our effort to decipher the role of photoreceptor synaptic ribbons in SV release. In the present study, we used mouse cone photoreceptor development as a model system to describe and compare evoked SV release at synapses containing ribbon-free or ribbon-occupied AZs. We used a combination of whole-cell patch-clamp recordings of cone photoreceptors and electron microscopy to analyze SV pool size, SV replenishment rate, domain organization of Ca^2+^ channels, and the Ca^2+^ sensitivity of SV release. We compared cone photoreceptors at P8-9 and P12-13 (predominantly ribbon-free AZs) with cone photoreceptors at >P30 (predominantly synaptic ribbon-occupied AZs) and found that the synaptic ribbon is a key regulator of the SV pool size, and increases the Ca^2+^- sensitivity of glutamate release.

## Materials and methods

### Animals

Male and female C57BL/6J (Jackson Laboratories, Bar Harbor, ME) and Tg (Rac3-EGFP) JZ58Gsat/Mmcd (Rac3-EGFP) mice were used in this study. Rac3–enhanced green fluorescent protein (EGFP) mice are of a C57BL/6 background and express EGFP in all cone photoreceptor cells (Gong et al., 2003; Regus-Leidig et al., 2014). The mice were obtained from the Mutant Mouse Regional Resource Center (MMRRC), a National Center for Research Resources (NCRR) -NIH-funded strain repository and were donated to the MMRRC by the National Institute of Neurological Disorders and Stroke–funded GENSAT BAC transgenic project. Mice were kept on a 12-h light/dark cycle. The adult mice group contained mice in the range of P30–P90 (>P30). All experiments were performed in compliance with the guidelines for the welfare of experimental animals issued by the Federal Government of Germany, and the University of Erlangen-Nürnberg.

### Slice preparation and electrophysiology

Mice were anesthetized with isoflurane (3%) and euthanized by cervical dislocation. The retina was removed from the eye, placed in Ames’ medium (Sigma-Aldrich, Munich, Germany), and cut into quarters. Then, the retina was mounted flat in 1.8% low melting agarose dissolved in Ames’ medium and the excess agarose was removed using a vibratome (Leica Microsystems, Wetzlar, Germany). Wholemount retinas were visualized using a 63× water immersion objective (Zeiss, Jena, Germany) on a fixed stage microscope (Zeiss Axio Examiner). To remove photoreceptor outer segments and expose photoreceptor cell bodies a sharp glass electrode was moved on the surface of the retina using the micromanipulator. Cone photoreceptors were identified using EGFP fluorescence in the Rac-EGFP mice. Whole-cell patch-clamp recordings were made targeting cone photoreceptor somas. Puff application experiments of glutamate (1 mM) or DL-threo-β-Benzyloxyaspartic acid (TBOA, 350 μM) were performed in horizontal retinal slices in Rac-EGFP mice where a cut was made through the outer plexiform layer using a vibratome. This slicing procedure was described previously in detail (Feigenspan and Babai, 2017). For the puff application, we used the VM8 Perfusion System (ALA Scientific Instruments, Inc., New York, USA). The application tube had a diameter of ∼75 μm and was positioned a few hundred micrometers away from the targeted cell. During the puff application experiments, P8–9 cone photoreceptor cell bodies and >P30 synaptic terminals were targeted with the recording electrode. Currents were recorded using an EPC-10 patch-clamp amplifier (Heka Elektronik, Lambrecht, Germany), low-pass filtered at 2.9 kHz using a built-in Bessel filter, and digitized at 10 kHz with the Patchmaster software (HEKA Elektronik GmbH, Reutlingen, Germany). All recordings were made at room temperature (22–24°C) under ambient light conditions. During recordings slices were continuously perfused (∼1 mL/min) with bubbled (95% O_2_/5% CO_2_) extracellular solution containing (in mM/L): 116 NaCl, 22.6 NaHCO_3_, 1.25 NaH_2_PO_4_, 2.5 KCl, 2 CaCl_2_, 1 MgCl_2_, 10 glucose, 5 HEPES, 1 ascorbic acid, and 2 sodium pyruvate, adjusted to pH 7.4). Patch pipettes were pulled from borosilicate glass (Sutter Instruments, Novato, CA) to a final resistance of 10–14 MΩ.

Glutamate transporter-associated anion current (I_*AGlu*_) recordings were performed using a cesium/thiocyanate-based intracellular solution which contained (in mM/L): 82.5 potassium thiocyanate (KSCN^–^), 30 Cs-gluconate, 13.3 Cs-Glutamate, 1.6 EGTA, 11 TEA-Cl, 11.6 HEPES, 3 Mg^2+^ -ATP, 1.8 Mg^2+^-GTP, 0.67 CaCl_2_, and 0.67 MgCl_2_ (pH 7.2). SCN^–^ is the most permeant of the chaotropic anions through transporter-associated anion conductance (Eliasof and Jahr, 1996). Recordings with an access resistance exceeding 60 MΩ were excluded. For most of the experiments, we used Ca^2+^ channel tail currents generated by brief, strong pulses (from < 0.5 to 5 ms, + 28 mV) returning to −60 mV holding potential, to trigger SV release. This protocol caused the strong activation of voltage-gated Ca^2+^-channels fast enough to separate Ca^2+^ and glutamate transporter activated conductances.

Capacitance measurements were made using the Sine + DC technique, the lockin extension of the EPC-10 amplifier (sine wave frequency 800 Hz, peak amplitude 30 mV). We blanked output measurements for 10 ms after the step stimulus and began measurements 10 ms later to circumvent any influence of gating charges and let time for the phase angle feedback circuitry to settle. Recordings were excluded that had an access resistance exceeding 40 MΩ or a holding current below –30 pA at V_*h*_ = –60 mV. Capacitance measurements were performed using a cesium-glutamate based intracellular solution (in mmol/L): 90 Cs-gluconate, 40 Cs-glutamate, 5 EGTA, 13 TEA-Cl, 10 HEPES, 2.5 Mg-ATP, 2.5 Mg-GTP, 1 MgCl2, 1 CaCl_2_ (pH 7.2).

Tonic and spontaneous I_*AGlu*_ events recorded from cone photoreceptors were analyzed using the Mini Analysis software (Synaptosoft, Fort Lee, NJ), where a minimum of 100 I_*AGlu*_ events per cell were collected. For spontaneous event analysis, amplitude histograms of I_*AGlu*_ events were fit to a multiple Gaussian function consisting of three Gaussian distributions. Events that were less than the mean + the standard deviation of the peak of the first Gaussian distribution were classified as single SV events. Traces were low-pass filtered with a cut-off frequency of 1 kHz for illustration. When stimulation protocols were used, a minimum of 45 s was given between stimulations.

### Electron microscopy

For conventional electron microscopy, specimens were treated as described elsewhere (Davison et al., 2022b). Retinas of C56BL/6J mice at P8, P10, P12, and P30 (*n* = 3 for each age group) were sequentially fixed in 4% PFA for 1 h, followed by fixation in 2.5% glutaraldehyde for 2 h at room temperature. Retinas were postfixed in 2% osmium tetroxide and 3% K_4_[Fe(CN)_6_] in 0.1 M cacodylate buffer for 1.5 h. After dehydration in rising EtOH concentrations (30–100%) and propylene oxide, retinas were embedded in Epon resin (Fluka, Buchs, Switzerland). For analysis, semithin sections were cut with an Ultracut E microtome (Reichert-Jung/Leica Biosystems, Nußloch, Germany). Finally, samples were contrasted with lead citrate and Uranyless (Delta Microscopies, Mauressac, France) in an automatic contrasting system (EM AC20, Leica Microsystems). Ultrathin sections were examined and photographed with an EM10 electron microscope (Carl Zeiss) equipped with a SC1000 Orius™ CCD camera (GATAN, Pleasanton, CA) in combination with the DigitalMicrograph 3.1 software (GATAN). For analysis, we used the dataset from our previous publication (Davison et al., 2022b), consisting of approximately 150 randomly photographed cone photoreceptor terminals per timepoint. For the quantification of SVs, we placed boxes of 300 nm × 200 nm in the cytosol at ribbon-occupied and at ribbon-free AZs and counted all SVs within this region of interest. Boxes in the cytosol were placed randomly and were only manually moved when the random location was obstructed by non-vesicular structures (e.g., mitochondria, postsynaptic cells, or free-floating ribbons). Ribbon-free AZs were identified by the arciform shape of photoreceptor AZs, as well as the presence of the typical arrangement of postsynaptic cells (two horizontal cell processes and one bipolar cell dendrite).

### Statistical analysis

**FIGURE 1:**
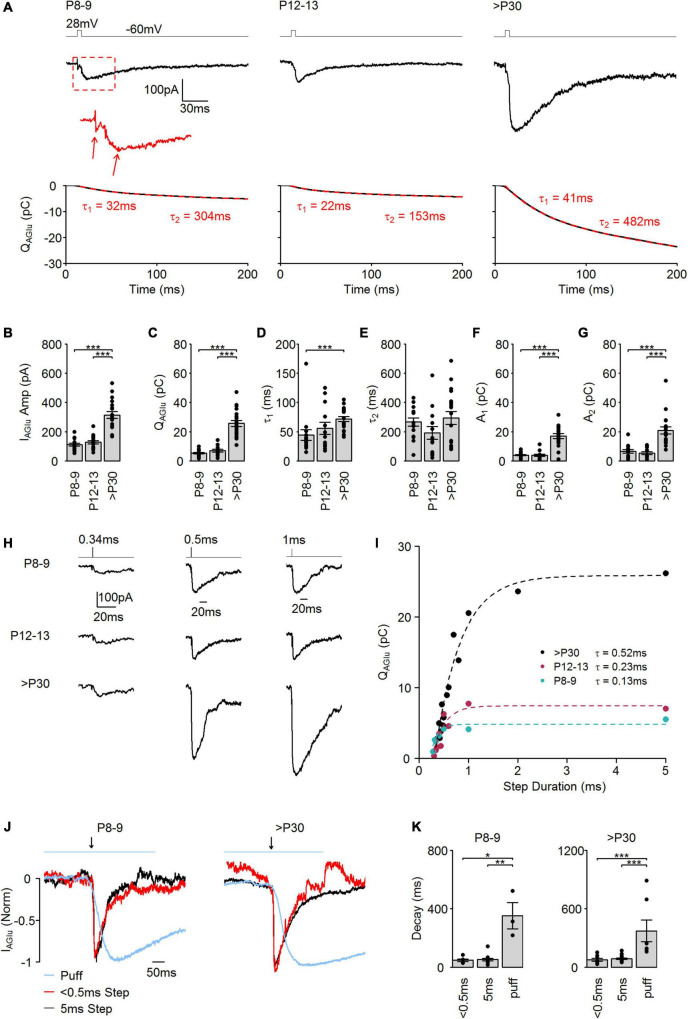
Evoked SV release in developing cone photoreceptors. **(A)** **Upper panel:** Representative evoked I_AGu_ events in response to a step depolarization (–60 to 28 mV, 5 ms) at the different developmental stages. **Bottom panel:** Black lines indicate the corresponding integral of the I_AGlu_ events. Dashed red lines indicate a double exponential fit to the integral. **(B)** Amplitude of evoked I_AGlu_ events (*p* < 0.001, *n* = 15–19 cells, one-way ANOVA test). *Post hoc* Tukey Test (P8–9 vs. P12–13: *p* = 0.821, P8–9 vs. >P30: *p* < 0.001, P12–13 vs. >P30: *p* < 0.001). **(C)** Charge transfer of evoked I_AGlu_ events (*p* < 0.001, *n* = 15–19 cells, Kruskal-Wallis test). *Post hoc* Mann–Whitney *U*-test (P8–9 vs. P12–13: *p* = 0.230, P8–9 vs. >P30: *p* < 0.001, P12–13 vs. >P30: *p* < 0.001). **(D)** Time constant (τ_1_) of the first release component (*p* = 0.001, *n* = 13–19 cells, Kruskal-Wallis test). *Post hoc* Mann–Whitney *U*-test (P8–9 vs. P12–13: *p* = 0.618, P8–9 vs. >P30: *p* < 0.001, P12–13 vs. >P30: *p* = 0.097). **(E)** Time constant (τ_2_) of the second release component (*p* = 0.156, *n* = 13–19 cells, Kruskal-Wallis test). **(F)** Amplitude of the first release component (A_1_). (*p* < 0.001, *n* = 13–19 cells, Kruskal-Wallis test). *Post hoc* Mann–Whitney *U*-test (P8–9 vs. P12–13: *p* = 0.590, P8–9 vs. >P30: *p* < 0.001, P12–13 vs. >P30: *p* < 0.001). **(G)** Amplitude of the second release component (A_2_) (*p* < 0.001, *n* = 13–19 cells, Kruskal-Wallis test). *Post hoc* Mann–Whitney *U*-test (P8–9 vs. P12–13: *p* = 0.590, P8–9 vs. >P30: *p* < 0.001, P12–13 vs. >P30: *p* < 0.001). **(H)** Representative I_AGlu_ events evoked by a step depolarization (–60 to 28 mV) of varying step duration at the different developmental ages. **(I)** Plot of charge transfer of I_AGlu_ events evoked by step depolarizations (–60 to 28 mV) of varied duration. Dashed lines: single exponential function fits (extra sum-of-squares *F*-test was used to compare the best fit with a two exponential fit function). **(J)** I_AGlu_ normalized by amplitude and aligned by rise-time evoked by a brief (<0.5 ms, 28 mV) or longer (5 ms, 28 mV) depolarizing step, or puff application of glutamate (1 mM, 500 ms) at premature (P8–9) and mature (>P30) developmental stages. Blue line on the top indicates period of glutamate puff and black arrow indicates time of depolarizing step. **(K)** Tau of a single exponential fit to I_AGlu_ event decay evoked by a brief (<0.5 ms, 28 mV) or longer (5 ms, 28 mV) depolarizing step, or puff application of glutamate (1 mM, 500 ms). P8–9: *p* = 0.019, *n* = 3–15 cells, Kruskal-Wallis test. *Post hoc* Mann–Whitney *U*-test (<0.5 vs. 5: *p* = 0.800, < 0.5 vs. puff: *p* = 0.036, 5 vs. puff: *p* = 0.007). P30: *p* < 0.001, *n* = 7–19 cells, Kruskal-Wallis test. *Post hoc* Mann–Whitney *U*-test (<0.5 vs. 5: *p* = 0.238, < 0.5 vs. puff: *p* < 0.001, 5 vs. puff: *p* < 0.001).

**FIGURE 2:**
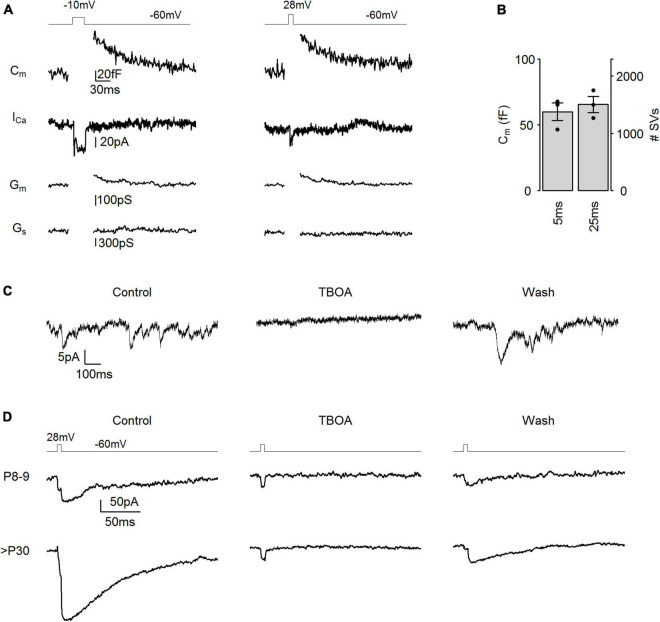
I_AGlu_ events as a reliable measure of SV release. **(A)** Representative membrane capacitance (C_m_) and Ca^2+^ current (I_Ca_) traces evoked by step depolarizations (**left panel**: –60 to –10 mV, 25 ms, **right panel**: –60 to 28 mV, 5 ms). G_m_: membrane conductance, G_s_: series conductance. **(B)** Membrane capacitance change in response to step depolarizations (*p* = 0.195, *n* = 3 cells, paired *t*-test). **(C)** Representative tonic I_AGlu_ events (V_h_ = –40 mV) in control conditions (Control), during puff application of 350 μM TBOA (TBOA), and following TBOA application (wash). **(D)** Representative I_AGlu_ events in response to a step depolarization (–60 to 28 mV, 5 ms) in control conditions (control), during puff application of 350 μM TBOA (TBOA), and following TBOA application (wash).

For normally distributed data, depending on the number of independent variables, a one or two-way ANOVA test was performed with the *post hoc* Tukey test for comparisons between individual groups. The assumption of normality was tested using the Shapiro–Wilk test. Statistical testing in non-normally distributed data was performed using the Kruskal–Wallis test with *post hoc* pairwise Mann–Whitney *U*-tests with adjustment of the *P*-value for multiple comparisons using the Benjamini and Hochberg method. The extra sum-of-squares *F*-test was used to determine whether a single or double exponential function fit data points the best. All data are presented as the mean ± standard error of the mean (SEM). *P*-values of the statistical significance were denoted on the figures by asterisks: non significance: no star, *P = 0.05, **P = 0.01, and ***P = 0.001. A minimum of 3 animals were used for each experiment apart from control experiments reported in Figures 1J,K, 2, where 1–3 animals were used.

## Results

### Measurement of evoked synaptic vesicle release in developing cone photoreceptors using I_AGlu_

In our previous study, we demonstrated that at early postnatal days, AZs in mouse cone photoreceptor terminals are predominantly ribbon-free, whilst in mature cone photoreceptors the majority of AZs are ribbon-occupied (Davison et al., 2022b). To investigate how AZ-attached synaptic ribbons regulate the kinetics and efficacy of SV release, we performed whole-cell patch-clamp recordings from cone photoreceptors at P8–9 (most AZs are ribbon-free), P12–13 (the period of eye-opening), and >P30 (most AZs are ribbon-occupied). Patch-clamp recordings were performed from cone photoreceptor cell bodies in wholemount retinas. To access the cell bodies, the outer segments were broken by moving a sharp glass pipette horizontally on the surface of the tissue. Cone photoreceptors were targeted using Rac3-EGFP mice, where cone but not rod photoreceptors express EGFP. We detected SV release by measuring glutamate transporter-associated anion currents (I_*AGlu*_). Glutamate is released from the synaptic terminal and taken up from the synaptic cleft by the presynaptic excitatory amino acid transporters (EAATs) (Kovermann et al., 2021). EAATs are located at photoreceptor terminals and their glutamate transport is coupled to anion channels, which generate a current proportional to the quantity of SVs exocytosed (Otis and Jahr, 1998; Palmer et al., 2003; Hays et al., 2020). We triggered SV release by a strong, brief stimulus (28 mV, 5 ms) from a –60 mV holding potential (V_*h*_), which resulted in typical evoked, EPSC-like, I_*AGlu*_ trajectories in all examined postnatal stages (Figure 1A). I_*AGlu*_ evoked by the voltage step stimulation contained two inward deflections (Figure 1A, arrows). As shown in previous work, the first deflection is produced by Ca^2+^-activated conductances. The second deflection is generated by EAAT-coupled anion currents and reflects SV release (Palmer et al., 2003). The measured I_*AGlu*_ amplitude (Figure 1B) and the charge transferred by I_*AGlu*_ = Q_*AGlu*_ (Figure 1A, bottom panels) were significantly higher at >P30 (Figure 1C), demonstrating a higher amount of SV release at >P30 than at P8–9 or P12–13. Dividing Q_*AGlu*_ by the number of AZs present at cone photoreceptor terminals during postnatal development (Davison et al., 2022b) (P8–9: ∼5 AZ/cone; P12–13: ∼8/AZ/cone; >P30: ∼13 AZ/cone) give rise to ∼1 pC of Q_*AGlu*_ at P8–9 and P12–13, and ∼2 pC of Q_*AGlu*_ at >P30. This indicates that during early postnatal developmental stages, when AZs at the cone photoreceptors are predominantly ribbon-free, the RRP of available SVs is smaller than at an adult stage when AZs are predominantly ribbon-occupied. Q_*AGlu*_ trajectories were best fitted with a double exponential in all age groups (Figure 1A, bottom panels, dashed red lines). The fast SV release component (τ_1_) was significantly slower at >P30 compared with P8–9 (Figure 1D). However, the slow release component (τ_2_) stayed similar throughout the examined developmental stages (Figure 1E). The amplitude of the exponential fit function was significantly higher in the >P30 group than at the pre-maturity stages (Figures 1F,G). The two kinetic components of Q_*AGlu*_ trajectories could be representative of the kinetics of SV release or the kinetics of glutamate clearance from the synaptic cleft (Bergles and Jahr, 1997). Consequently, we measured and compared Q_*AGlu*_, at different voltage step durations to measure if SV release occurs in one or two kinetic phases (Figure 1H). Interestingly, we found that the relationship between step durations and Q_*AGlu*_ was best fit with a single exponential function in all age groups with a tau value between ∼0.1 and 0.5 ms (Figure 1I), suggesting that SV release occurred from one cohort of primed SVs. Therefore, the two Q_*AGlu*_ components evoked by a brief, strong stimulus probably represent the kinetics of the presynaptic glutamate transporters at the cone photoreceptors. To test if Q_*AGlu*_ responses do not saturate during development, we compared I_*AGlu*_ event decays evoked by short (<0.5 ms, 28 mV) and longer (5 ms, 28 mV) depolarizing stimuli at P8–9 and >P30 age groups. In Figure 1J black and red traces illustrate examples of I_*AGlu*_ traces evoked by short and longer stimulation. Event decays did not show a significant difference between short and longer stimuli in P8–9 and >P30 age groups (Figure 1K), suggesting that the mechanism of glutamate clearance does not change during postnatal development and probably develops in parallel with SV release parameters. We also compared Q_*AGlu*_ responses evoked by voltage step (5 ms, 28 mV) and puff application of a high concentration of glutamate to investigate if EAAT saturation persists during voltage step responses. We puff-applied 1 mM (500 ms) glutamate onto cone photoreceptor terminals and recorded I_*AGlu*_ response (Figure 1J, blue traces). We found that 1 mM glutamate-evoked responses had significantly slower decays than those responses evoked by a depolarizing step (5 ms, 28 mV) in both developing (P8–9) and mature (>P30) cone photoreceptors (Figure 1K). These results demonstrate that voltage step-evoked I_*AGlu*_ events are not saturating presynaptic EAATs and consequently I_*AGlu*_ events are a reliable measurement of SV pool size in developing photoreceptors.

To trigger exocytosis of the entire RRP from cone photoreceptors, a longer but weaker test pulse (–10 mV and 25 ms) is typically applied (Babai et al., 2010; Bartoletti et al., 2010). To confirm that a 28 mV, 5 ms stimulus and a –10 mV, 25 ms stimulus can evoke equally large SV release, we performed capacitance measurements from adult cone photoreceptors (>P30) (Figure 2A). We found that capacitance jumps (ΔC_*m*_) triggered by both stimuli were not significantly different from each other (Figure 2B), indicating that the entire RRP pool was released in both cases. This suggests that voltage step stimuli (28 mV, 5 ms) applied in Figure 1A released the entire RRP. We calculated the size of the RRP from ΔC_*m*_ using a single SV capacitance of 43.7 aF, based on a single SV diameter of 37.3 nm (Regus-Leidig et al., 2014), which revealed a RRP of ∼1,400 SVs per cone photoreceptors (Figure 2B). To ensure that measured I_*AGlu*_ events (Figure 1A) were exclusively a result of glutamate reuptake, we applied 350 μM of the EAAT blocker TBOA by extracellular puff application and measured I_*AGlu*_ events at V_*h*_ = −40 mV when tonic SV release occurs due to the opening of voltage-sensitive Ca^2+^ channels in cone photoreceptors (Figure 2C). I_*AGlu*_ events ceased during TBOA application and I_*AGlu*_ events reappeared during the wash out period. Additionally, we puff applied 350 μM TBOA during voltage step stimulation (5 ms, 28 mV) and we found that the application of TBOA blocked the I_*AGlu*_ in both P8–9 and >P30 cone photoreceptors, exposing a small inward current equivalent to Ca^2+^ conductances (Figure 2D; Palmer et al., 2003). This suggests that, besides Ca^2+^ conductances, non-EAAT generated currents do not contribute to the evoked or tonic I_*AGlu*_ events and therefore are the results of synaptic activity. The observed Ca^2+^ conductance comprised 3.9 ± 1.5% (*n* = 6 cells) of the total area of the current trajectory and consequently had a minor impact on I_*AGlu*_ responses and thus on the SV release measurements.

**FIGURE 3:**
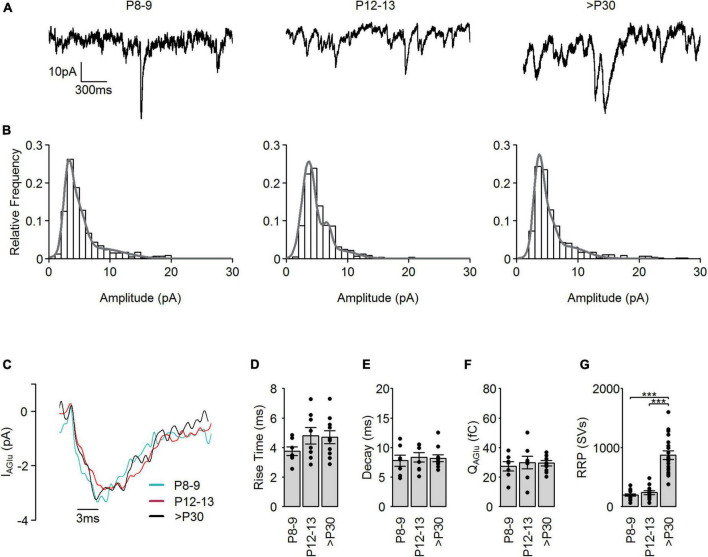
Spontaneous SV release in developing cone photoreceptors. **(A)** Representative spontaneous I_AGlu_ events recorded at V_h_ = –60 mV at the different developmental stages. **(B)** Amplitude histograms of spontaneous I_AGlu_ events (bin = 1 pA) at the different developmental stages. Gray line shows a fit of the distribution to a multiple Gaussian function. **(C)** Representative single SV I_AGlu_ events recorded at V_h_ = –60 mV at the different developmental stages. **(D)** 10–90% rise time of single I_AGlu_ events (*p* = 0.469, *n* = 7–10 cells, one-way ANOVA). **(E)** Tau of a single exponential decay of single I_AGlu_ events (*p* = 0.848, *n* = 7–10 cells, one-way ANOVA). **(F)** Charge transfer of single I_AGlu_ events (*p* = 0.853, *n* = 7–10 cells, one-way ANOVA). **(G)** RRP, calculated by dividing the charge transfer of evoked I_AGlu_ events by the mean charge transfer of single I_AGlu_ events for each developmental stage (*p* < 0.001, *n* = 11–16 cells, Kruskal-Wallis test). *Post hoc* Mann–Whitney *U*-test (P8–9 vs. P12–13: *p* = 0.42, P8–9 vs. >P30: *P* < 0.001, P12–13 vs. >P30: *p* < 0.001).

To test if the significantly higher Q_*AGlu*_ in the >P30 group (Figure 1C) was caused by the exocytosis of more SVs or an increase in the neurotransmitter (glutamate) content of the SVs during postnatal development, we measured spontaneous I_*AGlu*_ events at V_*h*_ = –60 mV (Figure 3). Example traces are illustrated in Figure 3A. Amplitude histograms of spontaneous I_*AGlu*_ events at V_*h*_ = –60 mV at all developmental stages were best fit with a multiple Gaussian function (Figure 3B), suggesting that spontaneous SV release events in mature and premature cone photoreceptors consist of single- and multivesicular SV release. In our previous study, we showed that multivesicular release is present during the whole postnatal development and that the synaptic ribbon likely plays a role in coordinating SVs in this process (Davison et al., 2022b). We classified those I_*AGlu*_ events that were less than the mean + standard deviation of the first Gaussian peak as single SV release events. The average amplitude of single I_*AGlu*_ events in all age groups was ∼3 pA (Figure 3C). We found no significant difference in the kinetics of single I_*AGlu*_ events, such as the rise time (Figure 3D), decay time (Figure 3E), or charge transfer (Figure 3F). This suggests that the higher I_*AGlu*_ charge transfer at >P30 was caused by an increase in the number of SVs in the RRP and not by increased glutamate content of the SVs. Therefore, we calculated the number of SVs in the RRP by dividing the charge transfer of the evoked SV release (Figure 1C) by the charge transfer of a single SV (Figure 3F). We found a pool size of ∼200 SVs in P8–9 and P12–13, which rose to more than 900 SVs at >P30 (Figure 3G) in cone photoreceptors. Dividing the estimated RRP (Figure 3G) by the number of AZs present at cone photoreceptor terminals (Davison et al., 2022b) resulted in ∼30 SVs/AZ in P8–9 and P12–13 and ∼70 SVs/AZ in >P30. Similarly, in adult mouse rod photoreceptors a RRP of 87 SVs was reported (Grabner and Moser, 2021). These results also support the hypothesis that the presence of a synaptic ribbon at the AZ increases the RRP.

### Synaptic vesicle organization at the active zones of developing cone photoreceptors

**FIGURE 4:**
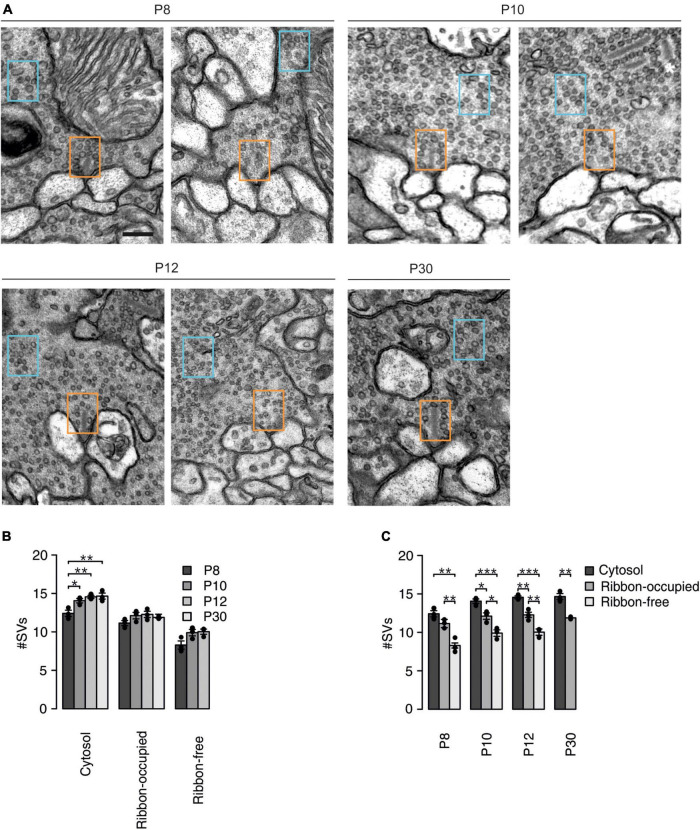
Postnatal changes in SV density in cone photoreceptors synaptic terminals. **(A)** Representative electron micrographs of ribbon-occupied (left) and ribbon-free (right) AZs at mouse cone photoreceptor terminals at the different developmental stages. Number of SVs was quantified in a 300 × 200 nm region in the cytosol (blue box) and at an empty or ribbon-occupied AZ (orange box). Scale bar: 200 nm. **(B)** Number of SVs in a 300 × 200 nm region in different synaptic regions at the different developmental stages (*p* < 0.001, *n* = 3 mice, two-way ANOVA). *Post hoc* pairwise Tukey tests of the cytosol group (P8 vs. P10: *p* = 0.033, P8 vs. P12: *p* = 0.008, P8 vs. P30: *p* = 0.006, P10 vs. P12: *p* = 0.718, P10 vs. P30: P = 0.603, P12 vs. P30: *p* = 0.997), ribbon-occupied AZ (P8 vs. P10: *p* = 0.258, P8 vs. P12: *p* = 0.164, P8 vs. P30: *p* = 0.457, P10 vs. P12: *p* = 0.986, P10 vs. P30: *p* = 0.963, P12 vs. P30: *p* = 0.848) and ribbon-free AZ (P8 vs. P10: *p* = 0.089, P8 vs. P12: *p* = 0.069, P10 vs. P12: *p* = 0.977). **(C)** Number of SVs in a 300 × 200 nm region in different synaptic regions at the different developmental stages (synaptic region: *p* < 0.001, *n* = 3 mice, two-way ANOVA). *Post hoc* pairwise Tukey tests of P8 group (ribbon-occupied AZ vs. ribbon-free AZ: *p* = 0.008, ribbon-occupied AZ vs. cytosol: *p* = 0.174: ribbon-free AZ vs. cytosol: *p* = 0.001), P10 group (ribbon-occupied AZ vs. ribbon-free AZ: *p* = 0.015, ribbon-occupied AZ vs. cytosol: *p* = 0.026: ribbon-free AZ vs. cytosol: *p* < 0.001), P12 group (ribbon-occupied AZ vs. ribbon-free AZ: *p* = 0.007, ribbon-occupied AZ vs. cytosol: *p* = 0.006: ribbon-free AZ vs. cytosol: *p* < 0.001) and P30 group (ribbon-occupied AZ vs. cytosol: *p* = 0.002).

It has been proposed that synaptic ribbons serve as a molecular scaffold for SVs, thus supporting neurotransmitter release (Matthews and Fuchs, 2010; Maxeiner et al., 2016). The ultrastructural examination of retinal cone photoreceptor synaptic terminals indicates that synaptic ribbons progressively anchor to the AZ during postnatal development (Davison et al., 2022b). Therefore, we examined how the appearance of synaptic ribbons alters SV distribution near the AZ and further away from the AZ, in the cytosol. Using electron microscopy, we imaged cone photoreceptor synaptic terminals during postnatal development (Figure 4A). At P30, we found very few AZs without a synaptic ribbon hence we did not include this group in the analysis. The number of SVs was counted in a 200 × 300 nm region at synaptic ribbon-free AZs, synaptic ribbon-occupied AZs, and in the cytosol some distance away from AZs. SVs were present throughout the synaptic terminals at all ages examined. We found a slightly but significantly lower cytosolic SV density at P8, which then stayed at a level of ∼14 SVs/0.06 μm^2^ from P10 to >P30 (Figure 4B). SV density stayed at a similar level during maturation both at ribbon-occupied and ribbon-free AZs (Figure 4B). Comparing SV densities at the investigated locations among the individual age groups showed significant differences (Figure 4C). At all developmental stages examined, we found the highest SV density in the cytosol, a lower density at ribbon-occupied AZs and the lowest density at ribbon-free AZs (Figure 4C). This indicates that mouse cone photoreceptors maintain a large pool of SVs in reserve, which does not depend on the presence of the synaptic ribbon at the AZ. Furthermore, these measurements show that the anchoring of synaptic ribbons significantly increases the number of SVs even when the synaptic ribbon reduced the available space at the AZ. The length of the synaptic ribbon at the adult cone photoreceptor synaptic terminal is around 1 μm (Davison et al., 2022b) and a SV in mouse photoreceptors has an average diameter of around 40 nm (Fuchs et al., 2014). Considering these measurements and that the first two rows of the SVs (∼100 nm) comprises the RRP (Thoreson, 2021) the EM measurements designate 100 SVs in the RRP [(1,000/40)*4] that agrees well with our physiological measurements (Figure 3G).

### Synaptic vesicle replenishment and recovery from synaptic depression in developing cone photoreceptors

**FIGURE 5:**
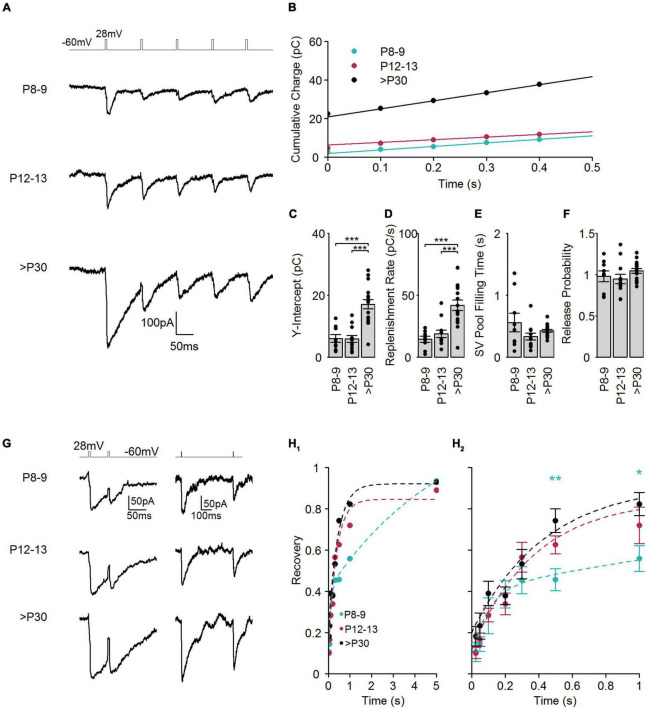
SV replenishment and recovery from synaptic depression during postnatal development. **(A)** Representative traces of I_AGlu_ events evoked by five sequential step depolarizations (–60 to 28 mV, 5 ms) at a frequency of 10 Hz at the different developmental stages. **(B)** Cumulative charge transfer of I_AGlu_ events at the different developmental stages. Lines are linear fits to the 0.2–0.4 ms region, which were extrapolated to the *Y*-axis. **(C)** RRP, calculated from the Y-intercept of the linear fits (*p* < 0.001, *n* = 9–17 cells, one-way ANOVA test). *Post hoc* Tukey Test (P8–9 vs. P12–13: *p* = 0.997, P8–9 vs. >P30: *p* < 0.001, P12–13 vs. >P30: *p* < 0.001). **(D)** The rate of SV replenishment calculated from the slope of the linear fits (*p* < 0.001, *n* = 9–17 cells, one-way ANOVA test). *Post hoc* Tukey Test (P8–9 vs. P12–13: *p* = 0.75, P8–9 vs. >P30: *p* < 0.001, P12–13 vs. >P30: *p* < 0.001). **(E)** The theoretical time to refill the RRP, calculated by dividing the RRP (C) by the replenishment rate (D). Significance was determined using a Kruskal-Wallis test (*p* = 0.133, *n* = 9–17 cells). **(F)** Release probability, RRP divided by the charge transfer of the first I_AGlu_ event of the train (*p* = 0.279, *n* = 9–17 cells, one-way ANOVA). **(G)** Representative examples of I_AGlu_ events evoked with a paired step depolarization (–60 to 28 mV for 5 ms) with a relatively short (50 ms) and long (500 ms) pulse interval at the different developmental stages. **(H_1_, _2_)** Plot of the recovery from synaptic depression (Q_AGlu_^2n*d*^/Q_AGlu_^1s*t*^). Paired-pulse intervals varied between 25, 50, 100, 200, 300, 500, 1,000, 5,000 ms. Dashed lines indicate exponential fits of recovery from synaptic depression. P12–13 and >P30 groups were best fit (extra sum-of-squares *F*-test) with a single exponential function whilst the P8–9 group was best fit with a double exponential function (P8–9: τ_1_ = 97 ms, τ_2_ = 5,667 ms, P12–13: τ = 364 ms, >P30: τ = 417 ms). **(H_2_)** shows an expanded view of recovery from synaptic depression between 0 and 1 s (25 ms: *p* = 0.283, 50 ms: *p* = 0.423, 100 ms: *p* = 0.315, 200 ms: *p* = 0.793, 300 ms: *p* = 0.637, 500 ms: *p* = 0.005, 1,000 ms: *p* = 0.0382, 5,000 ms: *p* = 0.88, one-way ANOVA test). *Post hoc* Tukey Tests for 500 ms interval (P8–9 vs. P12–13: *p* = 0.269, P8–9 vs. >P30: *p* = 0.004, P12–13 vs. >P30: *p* = 0.269). *Post hoc* Tukey Tests for 1,000 ms interval (P8–9 vs. P12–13: *p* = 0.305, P8–9 vs. >P30: *p* = 0.030, P12–13 vs. >P30: *p* = 0.529).

Synaptic information transfer at chemical synapses is limited by the ability of presynaptic cells to supply SVs as a source of neurotransmitters (Neher, 2010). Cone photoreceptors have a high demand for SVs due to the persistently high rates of SV release in the dark. Consequently, high rates of SV replenishment are essential to maintain persistent signaling. To investigate whether a change in the rate of SV replenishment occurs during the transition from primarily ribbon-free to ribbon-occupied cone photoreceptor AZs, we applied a train of five voltage steps (28 mV, 5 ms) with a frequency of 10 Hz to evoke I_*AGlu*_ events (Figure 5A). The SV replenishment rate was estimated by plotting the cumulative charge transferred by I_*AGlu*_ events against time (Figure 5B). A linear regression line was fitted to the last three points of the plot, where SV release is supposed to depend only on the replenishment rate (Schneggenburger et al., 1999; Kaeser and Regehr, 2017). Consequently, the Y-intercept of the fitted regression line corresponds to the size of the RRP. We found that the replenishment rate, similarly to the SV pool size, was significantly faster at >P30 than in the other age groups (Figures 5C,D). However, when we calculated the theoretical time taken to fill the entire SV pool, we found similar filling times in immature and mature cone photoreceptors (Figure 5E). This indicates that the mechanism(s) required to sustain SV replenishment to a 10 Hz stimulation perform at a similar efficiency during the whole postnatal cone photoreceptor development. We estimated the release probability by dividing the size of the RRP by the pool size of the first evoked I_*AGlu*_ event. This method was previously used to estimate release probability at photoreceptors (Bartoletti et al., 2010). Release probability showed no significant difference between all age groups measured (Figure 5F) and both mature and immature cone photoreceptors had a release probability close to one. High (close to 1) release probability was shown before for adult photoreceptors (Bartoletti et al., 2010). Altogether, these measurements suggest that the SV replenishment rate increases in the presence of a larger RRP during cone photoreceptor development and not by accelerating the refilling time of the RRP. The attachment of synaptic ribbons to AZs also does not appear to affect SV release probability during cone photoreceptor development.

Recovery from synaptic depression is thought to depend principally on the replenishment of SVs in the RRP. To investigate recovery from synaptic depression in developing cone photoreceptors, we applied paired stimuli (each 28 mV, 5 ms), with varying inter-pulse intervals (in ms: 25, 50, 100, 200, 300, 500, 1,000, 5,000) and measured I_*AGlu*_ events (Figure 5G). The recovery time constant was estimated by fitting an exponential function to the data points of the relative recovery (Figures 5H_1_,_2_). We found that at P8–9, recovery was best fit with a double exponential function having a time constant of ∼100 ms and ∼5.5 s, respectively. More mature cone photoreceptors (P12–13 and >P30) showed a single time constant of ∼ 400 ms. Comparing recovery time points among different age groups, we found that the recovery from synaptic depression was not significantly different among the examined age groups within the first 300 ms (Figure 5H_2_). However, the P8–9 group had significantly less recovery from depression at longer (500 ms and 1 s) paired-pulse intervals. The P12–13 group still has a relatively low percentage of ribbon-occupied AZs (Davison et al., 2022b), thus the recovery from synaptic depression in P12–13 cone photoreceptors probably does not depend on the attachment of synaptic ribbons to the AZs. The two kinetic recovery components at P8–9 most likely also do not originate from the presence of a synaptic ribbon at the AZ but rather from premature presynaptic handling of Ca^2+^ (Kirischuk et al., 2002; Garcia-Perez and Wesseling, 2008).

### Ca^2+^ dependence of synaptic vesicle release in developing cone photoreceptors

**FIGURE 6:**
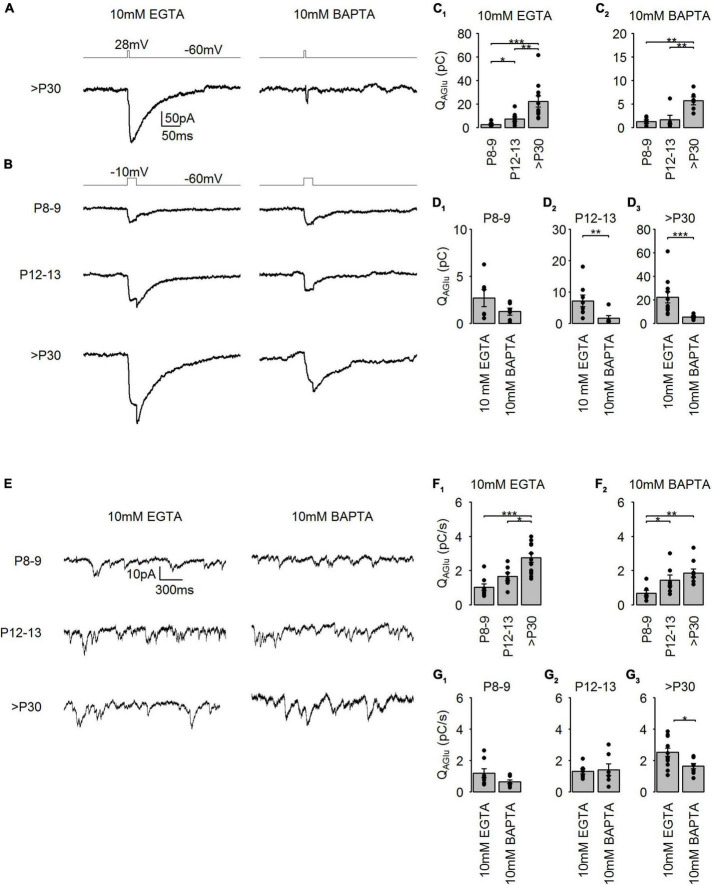
Developmental changes in Ca^2+^ channel domain organization at mouse cone photoreceptors. **(A)** Representative traces of I_AGlu_ events evoked by step depolarizations (–60 to 28 mV for 5 ms) in the presence of either 10 mM EGTA or 10 mM BAPTA at >P30. **(B)** Representative traces of I_AGlu_ events evoked by step depolarizations (–60 to –10 mV for 25 ms) in the presence of either 10 mM EGTA or 10 mM BAPTA at the different developmental stages. **(C_1_)** Charge transfer of I_AGlu_ evoked by step depolarizations (–60 to –10 mV for 25 ms) in the presence of 10 mM EGTA (*p* < 0.001, *n* = 6–11 cells, Kruskal-Wallis test). *Post hoc* Mann–Whitney *U*-test (P8–9 vs. P12–13: *p* = 0.043, P8–9 vs. >P30: *p* < 0.001, P12–13 vs. >P30: *p* = 0.004). **(C_2_)** Charge transfer of I_AGlu_ evoked by step depolarizations (–60 to –10 mV for 25 ms) in the presence of 10 mM BAPTA (*p* = 0.001, *n* = 6 cells, one-way ANOVA). *Post hoc* Tukey tests (P8–9 vs. P12–13: *p* = 0.726, P8–9 vs. >P30: *p* = 0.002, P12–13 vs. >P30: *p* = 0.005). **(D_1–3_)** Same data as in **(C_1_, _2_)** but data was compared in each age groups. P8–9: *p* = 0.189, *n* = 6 cells, unpaired *t*-test; P12–13: *p* = 0.008, *n* = 6–8 cells, Mann-Whitney *U*-test; >P30: *p* < 0.001, *n* = 6–11 cells, Mann-Whitney *U*-test. **(E)** Representative traces of I_AGlu_ events recorded from cone photoreceptors at V_h_ = –40 mV in the presence of 10 mM EGTA and 10 mM BAPTA, respectively. **(F_1_)** Rate of charge transfer of I_AGlu_ events in the presence of 10 mM EGTA (*p* < 0.001, *n* = 7–13 cells, one-way ANOVA test). *Post hoc* Tukey tests (P8-9 vs. P12–13: *p* = 0.283, P8–9 vs. >P30: *p* < 0.001, P12–13 vs. >P30: *p* = 0.014). **(F_2_)** Rate of charge transfer of I_AGlu_ events in the presence of 10 mM BAPTA (*p* = 0.005, *n* = 7–9 cells, Kruskal-Wallis test). *Post hoc* Tukey tests (P8–9 vs. P12–13: *p* = 0.039, P8–9 vs. >P30: *p* = 0.004, P12–13 vs. >P30: *p* = 0.211). **(G_1–3_)** Same data as in **(F_1_, _2_)** but data was compared in each age groups. P8–9: *p* = 0.121, 7–8 cells, Mann-Whitney *U*-test; P12–13: *p* = 0.799, 7 cells, unpaired *t*-test; >P30: *p* = 0.012, 9–13 cells, unpaired *t*-test.

SV release machinery is functionally coupled to voltage-gated Ca^2+^ channels, which are localized at the AZ. To determine the coupling distance between Ca^2+^ channels and the synaptic machinery during cone photoreceptor development, we measured SV release in the intracellular presence of 10 mM EGTA or 10 mM BAPTA (Figure 6A). EGTA is a Ca^2+^ chelator, which binds to Ca^2+^ with a slower on-rate than BAPTA does. Due to these kinetic differences, the comparison of the SV release in the presence EGTA and BAPTA is used to investigate the microdomain or nanodomain organization of the Ca^2+^ channels at the AZ (Neher, 1998; Wang and Augustine, 2014). The boundary splitting the two domains is suggested to be 50–150 nm (Eggermann et al., 2012). Since it has been proposed that synaptic ribbons play a role in organizing Ca^2+^ channels at the AZ (Maxeiner et al., 2016; Babai et al., 2019), we examined EGTA and BAPTA sensitivity of evoked SV release in developing cone photoreceptors. Brief stimuli (28 mV, 5 ms) evoked I_*AGlu*_ trajectories in the presence of 10 mM EGTA which were similar to I_*AGlu*_ measured using a 1.6 mM EGTA containing intracellular solution in adult cone photoreceptors (Figures 1A, 6A). However, SV release could not be evoked with a short, 5 ms long (28 mV) stimulus using a 10 mM BAPTA containing intracellular solution (Figure 6A). Consequently, we measured evoked SV release using a longer stimulus (–10 mV, 25 ms) (Figure 6B). SV release was estimated as the Q_*AGlu*_ measured from the end of the test pulse until I_*AGlu*_ returned to baseline (Grassmeyer et al., 2019).

We found that immature cone photoreceptors in the presence of either 10 mM EGTA or 10 mM BAPTA had a significantly smaller Q_*AGlu*_, indicating a reduced SV release (Figures 6C_1_, C_2_). Additionally, we found that the intracellular presence of 10 mM BAPTA caused a significant reduction of SV release at P12–13 and >P30 compared to 10 mM EGTA (∼77 and 75%, respectively) (Figures 6D_1–3_). This suggests that both during the eye-opening period and in maturity, voltage-sensitive Ca^2+^ channels are predominantly localized in a nanodomain organization close to the SV release machinery in cone photoreceptors. At P8–9, we could not detect a significant difference between measurements of evoked SV release in the presence of 10 mM EGTA and 10 mM BAPTA, suggesting that voltage-sensitive Ca^2+^ channels of immature cone photoreceptors are likely localized in microdomains with the SV release machinery. Only ∼30% of the synaptic ribbons are AZ-attached in P12–13 cone photoreceptors, which is different from >P30 where the percentage reaches ∼80% (Davison et al., 2022b). We thus conclude that AZ-attached synaptic ribbons likely do not play a direct role in Ca^2+^ channel localization into nanodomains at the AZ in the P12–13 group.

In darkness, cone photoreceptors are continuously releasing SVs because at this V_*m*_ (∼ –40 mV), voltage-gated Ca^2+^ channels are partially activated (Thoreson, 2021). In this condition, SVs located both closer and further from the AZ could contribute to the maintenance of tonic SV release (Innocenti and Heidelberger, 2008). Therefore, the distance between the Ca^2+^ channels and the tonically released SVs could be different from evoked SV release. To stimulate tonic SV release at developing cone photoreceptors, we held cone photoreceptors at V_*m*_ = –40 mV, a potential below the half-maximal activation of voltage-sensitive Ca^2+^ channels (Davison et al., 2022a,b), and measured I_*AGlu*_ events (Figure 6E). We found that at >P30, cone photoreceptors were capable of generating a significantly higher frequency of tonic SV release compared to immature cone photoreceptors (P8–9) in the presence of either 10 mM EGTA or 10 mM BAPTA (Figures 6F_1_,F_2_). Similar to evoked I_*AGlu*_ events, we detected a significant reduction in tonic SV release rates at >P30 when 10 mM BAPTA was present in the patch pipette compared to 10 mM EGTA, suggesting a nanodomain organization for tonic SV release (Figures 6G_1–3_). Nonetheless, tonic SV release rates were still relatively high (∼2 pC/s ≈ 67 SV/s) in >P30 cone photoreceptors in the presence of 10 mM BAPTA, suggesting that part of tonic SV release is not Ca^2+^-sensitive. In P8–9 and P12–13 groups, tonic SV release was similar in the presence of either 10 mM EGTA or 10 mM BAPTA, suggesting that tonic SV release may be a result of a microdomain organization at these developmental stages, or may be primarily Ca^2+^ insensitive. In general, it appears that at P8–9 a microdomain organization of Ca^2+^ channels controls evoked and tonic SV release. Interestingly, at the eye-opening period (P12–13) tonic SV release might be controlled by SVs further away from the AZ but evoked SV release is controlled by nanodomain organization. Moreover, in adult (>P30) cone photoreceptors, both tonic and evoked SV release take place in nanodomains.

**FIGURE 7:**
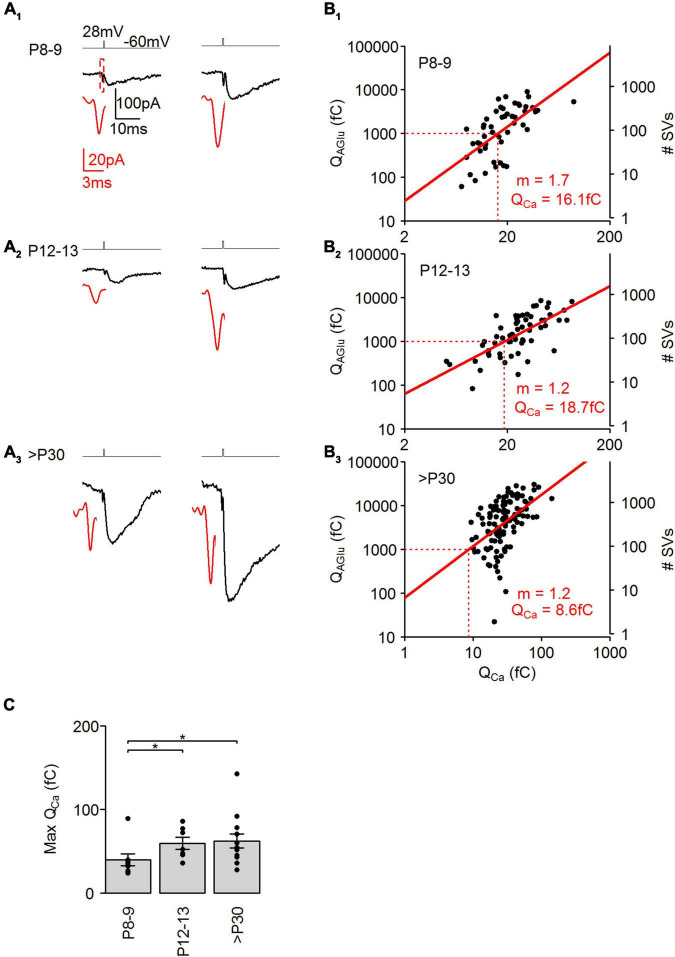
Postnatal development of Ca^2+^ dependence at cone photoreceptors. **(A_1–3_)** Representative I_AGlu_ events evoked by either a 0.38–0.56 ms (left) and 1 ms (right) step depolarization (from –60 to 28 mV). Black traces show the I_AGlu_ events and red traces show an expanded view of I_Ca_ preceding the I_AGlu_ events. **(B_1–3_)** Plot of the charge transfer of Q_Ca_ against the charge transfer of I_AGlu_ on a log-log axis. Red lines show linear regression fits of log transformed data. m = slope of fitted line. Red dashed lines indicate Q_Ca_ at Q_AGlu_ = 1,000 fC. Linear fits were significant at all developmental stages [(P8–9: y = 1.7x + 0.958, *r*^2^ = 0.426, *p* < 0.001, *n* = 51 events from 7 cells), (P12–13: y = 1.2x + 1.434, *r*^2^ = 0.472, *p* < 0.001, *n* = 61 events from 9 cells), (>P30: y = 1.2x + 1.897, *r*^2^ = 0.235, *p* < 0.001, *n* = 116 events from 13 cells)]. **(C)** Charge transfer of I_Ca_ for a step depolarization (1 ms, –60 to 28 mV) at the different developmental stages (*p* = 0.0264, Kruskal-Wallis Test). *Post hoc* Mann-Whitney *U*-test (P8–9 vs. P12–13: *p* = 0.043, P8–9 vs. >P30: *p* = 0.038, P12–13 vs. >P30: *p* = 1).

Domain organization of the Ca^2+^ channels at the AZ strongly influences the local Ca^2+^ concentration around the SVs, hereby influencing the Ca^2+^ dependency of SV release. In mature photoreceptor terminals, SV release is highly sensitive to Ca^2+^ with only a few Ca^2+^ ions required for the exocytosis of a single SV (Thoreson et al., 2004; Bartoletti et al., 2011). We thus investigated next, whether the Ca^2+^ sensitivity of SV release in cone photoreceptors changes during postnatal development. To evoke SV release with variable amplitudes, we used brief voltage steps (28 mV, 0.3–1 ms), which generated a relatively fast I_*Ca*_ isolated from the following I_*AGlu*_ trajectories in cone photoreceptors in all examined ages (Figures 7A_1–3_). Lengthening the voltage steps resulted in an increased charge transfer of I_*Ca*_ and I_*AGlu*_ from P8 to >P30 (Figures 7B_1–3_). The log-transformed I_*AGlu*_ and I_*Ca*_ charge data points were best fitted with a linear regression at all developmental ages, suggesting a proportional relationship between the two variables. SV release in adult photoreceptors is highly sensitive to Ca^2+^ and shows a relatively shallow relationship that is nearly linear (Thoreson et al., 2004). In our measurements, the slope of the fitted regression line was 1.7 at P8–9 (Figure 7B_1_), which suggests a Ca^2+^ cooperativity of ∼2. This result agrees with our findings shown in Figure 6, thus supporting a microdomain organization of Ca^2+^ channels at the AZ. At P12–13, the slope of the linear fit resulted in a Ca^2+^ cooperativity of ∼1 (Figure 7B_2_), which suggests the necessity of the opening of one (or a few) Ca^2+^ channels for the release of a single SV, indicating a nanodomain organization of Ca^2+^ channels at the AZ at the time of eye-opening. The slope of the fitted regression line was also ∼1 at >P30, which also indicates low Ca^2+^ cooperativity in mature mouse cone photoreceptors (Figure 7B_3_). Furthermore, we estimated the Ca^2+^ sensitivity of SV release by measuring at what Q_*Ca*_ value SV release reaches 1,000 fC of Q_*AGlu*_ (Figure 7B, dashed lines). We found that a higher QI_*Ca*_ value was required to trigger 1,000 fC of Q_*AGlu*_ at P8–9 and P12–13 groups (∼16–19 fC) than at the >P30 group (∼9 fC). This suggests that Ca^2+^ sensitivity is similar till eye-opening and then increases up to twofold in adulthood. Interestingly, the change in Ca^2+^ sensitivity temporally coincides with the appearance of synaptic ribbons at the AZ during development, suggesting a role of synaptic ribbons in regulating Ca^2+^ sensitivity of SV release at cone photoreceptors. We also determined maximal Ca^2+^ conductances by measuring the Q_*Ca*_ evoked by a 1 ms depolarizing step (Figure 7C). We found that QI_*Ca*_ increases from P8–9 to P12–13, but by P12–13 the size of the QI_*Ca*_ had already reached adult-like levels, suggesting that establishment of the voltage-sensitive Ca^2+^ channels at the AZ occurs prior to synaptic ribbon attachment. QI_*Ca*_ measurements also agree with previous I_*Ca*_ measurements in developing cone photoreceptors (Davison et al., 2022b).

## Discussion

Synaptic ribbons have long been known as a structural hallmark of photoreceptor and bipolar cell synapses in the retina, but their functional role in SV release is still unsolved. To better understand the function of synaptic ribbons in SV release, studies have used mouse models, which lack crucial presynaptic proteins, causing structural alterations or the complete absence of synaptic ribbons. For example, the removal of bassoon from photoreceptors causes the synaptic ribbon to not anchor to the AZ, which results in an altered synaptic transmission and an abnormal postsynaptic dendritic branching (Dick et al., 2003; Babai et al., 2019, 2021). The removal of piccolino, the ribbon synapse-specific splice variant of piccolo, results in an altered structure of attached synaptic ribbons and altered synaptic transmission (Muller et al., 2019). The removal of ribeye, the building block of the synaptic ribbon, leads to a complete absence of synaptic ribbons, causing impaired evoked and tonic neurotransmitter release. In ribeye-deficient synapses, the level of other proteins such as CtbP2 and RIM-BP1 are also changed (Maxeiner et al., 2016), which might influence the measurements. The synaptic proteins which are essential for fast and regulated exocytosis of SVs are already present in photoreceptors after P4/P5 (von Kriegstein and Schmitz, 2003). Additionally, it is proposed that the appearance of the basic synaptic fusion machinery precedes the formation of a complete ribbon synapse by several days (von Kriegstein and Schmitz, 2003). To decipher the functional role of the synaptic ribbon in SV release, we compared SV release at different postnatal stages (P8–9, P12–13, >P30), exploiting the transition from ribbon-free to ribbon-occupied AZs during the development of cone photoreceptor synapses.

### The synaptic ribbon as synaptic vesicle organizer

One of the main differences between conventional and ribbon-type synapses is that at conventional synapses SV release is tightly coupled to action potentials, whilst at ribbon synapses, membrane potential changes are converted gradually into SV release. However, there is also evidence for spike activity in adult cone photoreceptors (Davison et al., 2022a). Cone photoreceptors release SVs tonically at the dark membrane potential (Figure 6) during which continuous replenishment of SVs and a large reserve pool of SVs is required. We suggested previously that cone photoreceptor synaptic ribbons slow down tonic SV release and in this way might increase the RRP (Davison et al., 2022b). This finding agrees well with the proposed hypothesis that synaptic ribbons behave like a “safety belt” and stabilize mobile SVs near the AZ (Parsons and Sterling, 2003). Here, we provide physiological (Figure 1) and anatomical (Figure 4) evidence that the presence of synaptic ribbons increases the number of release-ready SVs at the synapse. We found that mature cone photoreceptors had an average RRP size of ∼900 SVs, whilst at P8–9 and P12–13, the pool size was ∼200 SVs, comprising a fourfold increase in the SV pool occurring after eye opening (P12–13). This difference is likely not caused by a developmental increase in Ca^2+^ influx, as Ca^2+^ currents already reach their maximum as early as P12–13 (Figure 7C; Davison et al., 2022b). We have previously reported that during this developmental period there is both an increase in the percentage attachment of synaptic ribbons (P8–9 = 24%, P12–13 = 33%, >P30 = 78%), as well as in the total number of synaptic ribbons present in cone photoreceptor terminals (P8–9 = 5.5, P12–13 = 7.3, >P30 = 10.6) (Davison et al., 2022b). This comprises a total number of attached synaptic ribbons per cone terminal of P8–9 = 1.32, P12–13 = 2.4, >P30 = 8.3. Consequently, the fourfold increase in SV pool size between P12 and >P30 coincides well with a ∼ 3.5-fold increase in the number of attached synaptic ribbons, demonstrating that developmental changes in SV pool size coincide temporally and quantitatively with postnatal increases in synaptic ribbon attachment. This suggests that the synaptic ribbon regulates the pool of SVs available for evoked SV release. This agrees well with previous reports where SVs associated with synaptic ribbons are exocytosed in response to a strong stimulus (Lenzi et al., 2002; Bartoletti et al., 2010). Moreover, fluorescence-activated laser inactivation and FM-dye fluorescence techniques have proved that SVs on synaptic ribbons contribute to SV release upon depolarization (LoGiudice et al., 2008; Snellman et al., 2011). Additionally, the deletion of RIBEYE from ribbon synapses significantly reduces the number of SVs at the AZ and within the RRP (Maxeiner et al., 2016; Grabner and Moser, 2018). Interestingly, we found in cone photoreceptors that SV density was higher in the cytosol than at the AZ during early postnatal development and also in adulthood (Figure 4). Cytosolic SVs typically form the reserve SV pool and might contribute to the replenishment processes. In cone photoreceptor terminals, ∼50–85% of SVs are mobile, which is higher than the mobility of the SVs tethered to the ribbon (∼20%) (Rea et al., 2004; Zenisek et al., 2004). Consequently, ribbon-tethered SVs are not in dynamic equilibrium with cytoplasmic SVs. Photoreceptors probably need a large and fast-releasing SV pool to support continuous and evoked SV release and this is provided from the cytosol and released through synaptic ribbons.

### Synaptic vesicle release in developing cone photoreceptors

SVs at the photoreceptor synapse are believed to be released from different SV pools with faster and slower release kinetics (Thoreson, 2021). We found that the fast, transient component, called the RRP, was released with a tau value of a few hundred microseconds and consisted of one kinetic component (Figure 1). Our measurements show that the RRP of an adult mouse cone photoreceptor consists of ∼70 SVs/ribbon. In comparison, in salamander cone photoreceptors, a common model of photoreceptor physiology, the RRP consists of ∼15–20 SVs/ribbon and release occurs within a few milliseconds, measured by paired recordings (Thoreson, 2007). Moreover, mouse rod photoreceptors consist of a RRP of 87 SVs/ribbon and release occurs with a tau of 0.4 ms measured by capacitance measurements (Grabner and Moser, 2021). Interestingly, the smaller tau values of the release kinetics in P8–9 and P12–13 groups suggest that the AZ-attached synaptic ribbon slows down the release of the RRP (P8–9: 130 μs vs. >P30: 520 μs). This agrees well with the increasing time constant (τ_1_) value of Q_*AGlu*_ (Figure 1) and our previous findings, where we used a bassoon mutant or juvenile synapse model system to examine ribbon-free synapses (Babai et al., 2021; Davison et al., 2022b). Our results support the hypothesis that the synaptic ribbon might behave like a safety belt in controlling SV release.

### Replenishment at developing cone photoreceptors

Evidence for the role of the synaptic ribbon in controlling SV replenishment is contradictory. It has been suggested that synaptic ribbons may aid replenishment, but also that synaptic ribbons may slow it down (Parsons et al., 1994; Jackman et al., 2009; Babai et al., 2019). We found that the replenishment rate during a 10 Hz stimulus was significantly higher in adult cone photoreceptors compared with younger ages (Figure 5). This corresponds well with the appearance of AZ-attached synaptic ribbons during development. Notably, the calculated filling time of the RRP was not different among the different developmental groups. This suggests that (i) the increased replenishment rate at >P30 was due to the increased RRP size and (ii) the rate of refilling of the RRP is not dependent on the ribbon attachment to the AZ. The recovery from synaptic depression is strongly controlled by the availability of release-ready SVs, thus by the rate of SV replenishment (Neher, 2010). In inner hair cell ribbon synapses, recovery from paired-pulse depression showed no difference in the presence or absence of synaptic ribbons (Becker et al., 2018; Jean et al., 2018). We only found a difference in recovery time between P8–9 and >P30 at 500 ms and 1 s indicating that the synaptic ribbon might not play a critical role in recovery processes in cone photoreceptors. SV replenishment is shown to be Ca^2+^-dependent in cone photoreceptors (Babai et al., 2010) and Ca^2+^ currents are comparable between P12–13 and adult ages but are significantly lower at P8–9 (Davison et al., 2022b). Thus, the level of Ca^2+^ is rather the rate limiting factor in the process of SV replenishment.

### The role of Ca^2+^ in developing cone photoreceptors

There is a tight temporal and spatial relationship between Ca^2+^ influx and SV release, meaning that small alterations in presynaptic Ca^2+^ levels significantly affect SV release. The comparison of Ca^2+^ influx and SV release in developing cone photoreceptor synapses showed a less linear relationship at P8–9 with a Ca^2+^ cooperativity of ∼2 and a more linear relationship at P12–13 and >P30 with a Ca^2+^ cooperativity of ∼1 (Figure 7). A linear relationship may be expected if the exocytosis of a single SV is triggered by nearby Ca^2+^ channels (Brandt et al., 2005). Conventional synapses typically show a non-linear relationship with a slope value of 3–5 (Augustine et al., 1985). Our evoked SV release measurements with slow and fast Ca^2+^ buffers show that cone photoreceptor synapses have a microdomain organization at P8–9 but a nanodomain organization at P12–13 and >P30 (Figure 6). At P12–13, similar to P8–9, only ∼30% of the AZs are ribbon-occupied (Davison et al., 2022b), thus it seems that the attachment of the synaptic ribbon at the AZ does not trigger the formation of nanodomains in cone photoreceptors. However, the Ca^2+^ sensitivity of SV release might be controlled by synaptic ribbons as they show similar values at P8–9 and P12–13 but an increased value at >P30, which is similar to the timing of synaptic ribbon attachment to the AZ during development. Synaptic ribbons might create a barrier for Ca^2+^ diffusion thereby increasing local Ca^2+^ concentrations for release-ready SVs. Similarly, mouse inner hair cells showed an increase in efficiency and an increase in the linearity of the Ca^2+^ dependence of exocytosis during development (Johnson et al., 2005). Interestingly, we found that the release probability, the ratio between the number of SVs released in the first stimulus and the RRP, was similar during postnatal development (Figure 5). This suggests that the synaptic ribbon does not influence the release probability of SVs in cone photoreceptors. This could indicate that a constantly high release probability is necessary both during development, e.g., to facilitate synapse formation (Andreae and Burrone, 2014) and in adulthood for proper visual information coding.

The synaptic machinery of cone photoreceptors must function unceasingly for the faithful transmission of visual information. Here, we studied ribbon-free and ribbon-occupied AZs in developing cone photoreceptors in order to decipher the role of the synaptic ribbon in SV release. Based on the presented experiments we propose that cone photoreceptor synaptic ribbons do not organize the voltage-sensitive Ca^2+^ channels into nanodomains or control SV release probability. However, the synaptic ribbon increases SV density at the AZ, increases the RRP for evoked SV release, aids SV replenishment by increasing the readily releasable SV pool size without changing the refilling rate and increases the Ca^2+^-sensitivity of the release machinery.

## Data availability statement

The raw data supporting the conclusions of this article will be made available by the authors, without undue reservation.

## Ethics statement

This animal study was reviewed and approved by the Federal Government of Germany, and the University of Erlangen-Nürnberg.

## Author contributions

NB contributed to the conceptualization, wrote the manuscript, and acquired resources and funding. NB and JB designed the research. AD, KG, and NB performed the experiments. AD and KG analyzed the data. JB, AD, and KG edited the manuscript. All authors contributed to the article and approved the submitted version.
